# Luspatercept versus mitapivat for non‐transfusion‐dependent β‐thalassemia: Dare to compare?

**DOI:** 10.1002/hem3.70165

**Published:** 2025-07-13

**Authors:** Khaled M. Musallam, Sujit Sheth, Maria Domenica Cappellini, Kevin H. M. Kuo, Antonis Kattamis, Yesim Aydinok, Vip Viprakasit, Ali T. Taher

**Affiliations:** ^1^ Center for Research on Rare Blood Disorders (CR‐RBD) and Thalassemia & Sickle Cell Center Burjeel Medical City Abu Dhabi United Arab Emirates; ^2^ Department of Public Health & Epidemiology Khalifa University Abu Dhabi United Arab Emirates; ^3^ Department of Pediatrics, Division of Hematology/Oncology Weill Cornell Medicine New York New York USA; ^4^ Unit of Medicine and Metabolic Disease Fondazione IRCCS Ca' Granda Ospedale Maggiore Policlinico Milan Italy; ^5^ Department of Medicine, Division of Hematology University of Toronto Toronto Ontario Canada; ^6^ Scarborough Health Network Scarborough Ontario Canada; ^7^ First Department of Pediatrics National and Kapodistrian University of Athens Athens Greece; ^8^ Department of Pediatric Hematology Ege University Medical School Izmir Turkey; ^9^ Department of Pediatrics & Thalassemia Center, Faculty of Medicine, Siriraj Hospital Mahidol University Bangkok Thailand; ^10^ Department of Internal Medicine American University of Beirut Medical Center Beirut Lebanon

There is now ample evidence that untreated anemia in patients with non‐transfusion‐dependent β‐thalassemia (NTDT) is associated with significantly increased risks of morbidity and mortality, challenging previous management strategies relying largely on conservative observation.^1^ Hemoglobin levels <10 g/dL are now used to indicate the need for intervention, and increases ≥1 g/dL are believed to be clinically meaningful based on data from observational studies.^2^, ^3^, ^4^, ^5^ The key challenge has been the lack of options beyond regular transfusions to treat anemia in NTDT, the latter being associated with secondary iron overload precluding its wide, long‐term application.^2^ Repurposing of drugs such as hydroxycarbamide was largely unsuccessful in NTDT.^6^ Splenectomy, which was once a common choice, has now become obsolete owing to increased risks of infections and thrombotic events.^2^ A new era of novel disease‐modifying therapeutics development has thus emerged in the past 10 years to address the unmet need of treating anemia in NTDT patients.

Several novel agents targeting hepcidin dysregulation have been evaluated for their effect on both iron overload and ineffective erythropoiesis or red cell survival, based on data from animal studies indicating a bidirectional relationship between both pathophysiologic mechanisms. However, results from clinical trials did not echo these findings, and most such programs have been terminated.^7^, ^8^ By targeting ineffective erythropoiesis via different modes of action, two novel agents “made it through” to late development as treatment options for anemia in NTDT. Luspatercept is a recombinant fusion protein made of a modified extracellular domain of the human activin receptor Type IIB fused to the Fc domain of human IgG1. These in turn bind to select transforming growth factor β superfamily ligands, block SMAD2/3 signaling, and enhance erythroid maturation during late‐stage erythropoiesis. Mitapivat is a first‐in‐class oral, small‐molecule, allosteric activator of the red blood cell‐specific form of pyruvate kinase that increases ATP levels and mitigates oxidative damage during erythropoiesis. Both agents have been shown to improve red cell survival and reduce hemolysis and/or ineffective erythropoiesis in β‐thalassemia mouse models.^9^, ^10^, ^11^, ^12^, ^13^, ^14^, ^15^ Luspatercept has been evaluated in adult patients with NTDT in the BEYOND trial and is now approved in Europe since 2023 (but not the United States) for this indication.^16^ Mitapivat has also been evaluated in adult patients with NTDT in the ENERGIZE trial, which recently met its endpoints, and is awaiting regulatory approvals and marketing authorization.^17^, ^18^ With no head‐to‐head comparison trial on the horizon and considering potential treatment choices (between the two agents) and that patient prioritization may become difficult for countries with limited access and affordability, we herein provide an indirect comparison of development and observed treatment effects based on available results from the BEYOND (NCT03342404)^16^, ^19^, ^20^, ^21^, ^22^ and ENERGIZE (NCT04770753)^17^, ^18^, ^23^ trials (Table 1), along with our expert insights.

**Table 1:** Key data from the BEYOND and ENERGIZE trials.^16^, ^17^, ^18^, ^19^, ^20^, ^21^, ^22^, ^23^

	BEYOND (luspatercept)	ENERGIZE (mitapivat)
Design	Phase 2, randomized (2:1), placebo‐controlled, double‐blind	Phase 3, randomized (2:1), placebo‐controlled, double‐blind
Eligibility		
Age	≥18 years	≥18 years
Thalassemia subtype	β‐Thalassemia	β‐ or α‐thalassemia
NTDT definition	0–5 RBC units during the 24 weeks before randomization Transfusion‐free for at least ≥8 weeks before randomization	≤5 RBC units during the 24 weeks before randomization No RBC transfusions ≤8 weeks before informed consent and no RBC transfusions during the screening period
Baseline Hb level	≤10 g/dL	≤10 g/dL
Stratification	Baseline Hb < 8.5 or ≥8.5 g/dL NTDT‐PRO T/W domain score ≥3 or <3	Baseline Hb ≤ 9.0 or 9.1–10.0 g/dL β‐Thalassemia or α‐thalassemia
Duration		
Double‐blind period	48 weeks	24 weeks
Open‐label extension	Up to 5 years (with roll‐over to long‐term follow‐up study)	Up to 5 years
Treatment		
Administration, dose	Subcutaneous every 3 weeks, 1–1.25 mg/kg	Oral twice daily, 100 mg
Dose modifications based on Hb response	Dose delay if Hb reaches >11.5 g/dL Dose reduction if Hb increased by >2 g/dL (from previous dose)	Dose reduction if Hb reaches >ULN (by sex)
Enrollment		
Planned	100 luspatercept 50 placebo	114 mitapivat 57 placebo
Actual	96 luspatercept (all received ≥1 dose) 49 placebo (all received ≥1 dose)	130 mitapivat (129 received ≥1 dose) 64 placebo (63 received ≥1 dose)
Baseline characteristics (treatment arm)		
Age (years)	Median: 39.5 (IQR: 28.0–48.5)	Mean: 42.4 (range: 19–68)
Females, n (%)	56 (58)	84 (65)
Thalassemia subtype, n (%)	β‐Thalassemia: 96 (100)	β‐Thalassemia: 88 (68) α‐Thalassemia: 42 (32)
Baseline Hb (g/dL)	Median: 8.2 (IQR: 7.4–9.2)	Mean: 8.3 (SD: 1.1)
Primary endpoint		
Endpoint definition	Proportion with mean Hb increase of ≥1.0 g/dL from baseline over a continuous 12‐week interval (Weeks 13–24), in the absence of RBC transfusions	Proportion with mean Hb increase of ≥1.0 g/dL from baseline from Week 12 through Week 24
Achieved, n (%)	Luspatercept: 74 (77) Placebo: 0 (0) P < 0.0001	Mitapivat: 55 (42.3) Placebo: 1 (1.6) P < 0.0001
Response by thalassemia subtype (treatment arm), n/N (%)	‐	β‐Thalassemia: 45/88 (51.1) α‐Thalassemia: 10/42 (23.8)
Response by baseline Hb strata (treatment arm), n/N (%)	Hb < 8.5 g/dL: 40/55 (72.7) Hb ≥ 8.5 g/dL: 34/41 (82.9)	Hb ≤ 9.0 g/dL: 45/95 (47.4) Hb 9.1–10.0 g/dL: 10/35 (28.6)
LSM change from baseline in Hb (treatment arm)	1.48 g/dL (SE: 0.07) [Weeks 13–24]	0.86 g/dL (95% CI: 0.73–0.99) [Weeks 12–24]
Change in PRO (key secondary endpoint)		
Tool	NTDT‐PRO T/W domain Decrease = improvement Score ≥ 3 = symptomatic Reduction ≥ 1 = clinically meaningful	FACIT‐Fatigue scale Increase = improvement MWPC ≥ 4.5
Endpoint definition	Change from baseline in mean NTDT‐PRO T/W domain score over a continuous 12‐week interval (Weeks 13–24)	Change from baseline in mean FACIT‐Fatigue scale score from Week 12 through Week 24
Baseline score	Luspatercept: median 4.3 (IQR: 2.4–5.8), score <3: 31% Placebo: median 4.1	Mitapivat: mean 36.1 Placebo: mean 36.41
LSM change from baseline in score	Luspatercept: −0.68 Placebo: −0.20 LSM difference: −0.48 P = 0.092	Mitapivat: 4.85 (MWPC ≥ 4.5: 36.2%) Placebo: 1.46 LSM difference: 3.40 P = 0.0026
Other PRO/functional changes	Data available for core and extension periods Greater changes in NTDT‐PRO T/W for luspatercept during Weeks 37–48 and in patients with baseline score ≥ 3, but not statistically significant against placebo Changes in NTDT‐PRO T/W domain score correlated with changes in Hb Durable improvements in NTDT‐PRO T/W with improvements in PGIC up to 96 weeks of treatment Improvements in FACIT‐Fatigue consistently across visits during the long‐term follow‐up period	Data available for core period Patients in the mitapivat arm had greater improvements in the 6MWT than those in the placebo arm at Week 24 A higher proportion of patients in the mitapivat arm reported improvements in fatigue as per PGIC versus those in the placebo arm at Weeks 12, 16, 20, and 24
Changes in biomarkers	Improvement in reticulocytes (without increases in fetal Hb) Reductions in spleen volume No changes in indirect bilirubin and LDH Improvement in serum ferritin in Hb responders	Improvements in reticulocyte percentage and erythropoietin Improvements in indirect bilirubin and LDH
Most common AEs (≥10% in treatment arm)	Headache, arthralgia, back pain, prehypertension, upper respiratory tract infection, hypertension, oropharyngeal pain, pharyngitis, cough, diarrhea, fatigue, influenza‐like illness, pain in extremity, pyrexia, asthenia, influenza irregular menstruation, toothache, insomnia, myalgia, gastroenteritis, and iron overload	Headache, initial insomnia, nausea, and upper respiratory tract infection

Abbreviations: 6MWT, 6‐minute walk test; AE, adverse event; CI, confidence interval; FACIT, functional assessment of chronic illness therapy; Hb, hemoglobin; IQR, interquartile range; LDH, lactate dehydrogenase; LSM, least‐squares mean; MWPC, meaningful within‐person change; NTDT, non‐transfusion‐dependent β‐thalassemia; NTDT‐PRO, non‐transfusion‐dependent β‐thalassemia‐patient‐reported outcome; PGIC, patient global impression of change; RBC, red blood cell; SD, standard deviation; SE, standard error; T/W, tiredness/weakness; ULN, upper limit of normal.

ENERGIZE was a larger Phase 3 trial, which overrecruited, compared to the Phase 2 BEYOND trial, which had challenges to reach planned recruitment targets, although without affecting planned statistical analyses or power calculations for primary and key secondary endpoints. However, one third of patients in ENERGIZE had α‐thalassemia (hemoglobin H disease) who were not included in the BEYOND trial (ENERGIZE was the first pivotal study to recruit α‐thalassemia), so both trials may be assumed relatively similar in size for β‐thalassemia assessment. Still, although ENERGIZE randomization was stratified by thalassemia genotype, comparison of main results from BEYOND with results for the β‐thalassemia subgroup in ENERGIZE should be made with caution. Both trials were randomized (2:1), double‐blind, and placebo‐controlled. Similar eligibility criteria were also used for the definition of transfusion independence, and a hemoglobin level of ≤10 g/dL was needed for inclusion. Both age and baseline hemoglobin levels were quite similar between trials. The core double‐blind treatment period for ENERGIZE was 24 weeks compared to 48 weeks in BEYOND, the latter also having available data from longer term open extension.^16^, ^17^, ^18^

For the primary endpoint of erythroid response, a greater proportion of patients achieved a mean hemoglobin increase from baseline by ≥1 g/dL (at Weeks 12/13–24) in patients in BEYOND than ENERGIZE (77% vs. 42.3%), with both being significantly greater than response observed in placebo; while noting that in ENERGIZE, response in patients with β‐thalassemia was 51.1%.^16^, ^17^, ^18^ Mean changes in hemoglobin from baseline to this visit interval were also greater in BEYOND than ENERGIZE (1.48 g/dL for luspatercept and 0.86 g/dL for mitapivat including both α‐ and β‐thalassemia). Two factors are important to consider in the interpretation of changes in hemoglobin. In the ENERGIZE trial, hemoglobin values that were within 8 weeks from a transfusion event during the trial were excluded from the primary endpoint analysis.^17^, ^18^ In BEYOND, only values within 21 days of a transfusion event were excluded.^16^ Whether this could have affected hemoglobin response rates is unclear, although the impact is expected to be minimal considering only a few patients on the treatment arms required transfusions. On the contrary, luspatercept dose modifications in BEYOND necessitated dose reduction or delay based on hemoglobin response (dose delay if hemoglobin reaches >11.5 g/dL and dose reduction if hemoglobin increased by >2 g/dL from the previous dose).^16^ For mitapivat, dose reductions were only mandated if patients' hemoglobin reached higher than the upper limit of normal.^17^, ^18^ This means that luspatercept's overall effect on mean hemoglobin change may have been limited by dose modifications.

A greater proportion of patients achieved a response in the lower versus higher baseline hemoglobin strata on mitapivat, whereas the opposite was observed on luspatercept.^16^, ^17^, ^18^ Whether this implies that mitapivat has a stronger impact in patients with severe anemia cannot be fully elucidated, and this may have been driven by the response rate in α‐thalassemia patients who commonly have higher hemoglobin values (data not reported).

When it comes to the assessment of patient‐reported outcomes (PROs, key secondary endpoint in both trials), we notice that the tides have turned. BEYOND did not meet the key secondary endpoint of a change in a newly developed tool, the NTDT‐PRO, in its tiredness/weakness (T/W) domain. The NTDT‐PRO is a six‐item questionnaire intended to measure the most relevant and important anemia‐related symptoms in the 24 h before administration. The six items assess tiredness (lack of energy, two items), weakness (lack of strength, two items), and shortness of breath (two items) when doing and when not doing physical activity. Each item uses an 11‐point numerical rating scale ranging from 0 (no symptoms) to 10 (extreme symptoms). Responses to the NTDT‐PRO can be used to derive T/W and shortness of breath domain scores. Although the NTDT‐PRO was specifically developed and validated for NTDT, this was the first time it was used in the context of a clinical trial. Whether this limited learning curve has affected outcomes assessment or whether this was driven by one third of patients being already asymptomatic at baseline is not clear.^16^ Nonetheless, patients on luspatercept had greater improvements in NTDT‐PRO than those on placebo, and the magnitude of difference was more prominent in patients who had a hemoglobin response, were symptomatic at baseline, and had longer follow‐up.^16^, ^19^ It is worth noting that fatigue was reported as an adverse event in around 13% of patients treated with luspatercept in both the BEYOND and the BELIEVE trial (transfusion‐dependent thalassemia), and it seems to occur irrespective of hemoglobin levels.^16^, ^24^ On the contrary, mitapivat showed significantly greater improvement over placebo in the functional assessment of chronic illness therapy (FACIT)‐Fatigue scale, an established tool for the assessment of PRO in patients with various types of anemia. Patients also had clinically meaningful improvements on other functional measures like the 6‐minute walk test.^17^, ^18^ Whether mitapivat is exerting these effects through amelioration of anemia or through other mechanisms that are “energizing” patients is yet to be determined.

Both agents seemed to be well‐tolerated in the trials, and common adverse events may not necessarily help with differentiation.^16^, ^17^, ^18^ Two concerns which have been raised from the longer term follow‐up of β‐thalassemia patients on luspatercept include the potential for worsening extramedullary hematopoietic paraspinal masses and some increased risk for thrombosis in patients with additional risk factors (e.g., splenectomy). These were seen at a higher rate in treated patients than in placebo, and thrombosis was more commonly observed in the BELIEVE trial (transfusion‐dependent patients) than BEYOND.^16^, ^24^, ^25^ Nonetheless, individuals with NTDT are more likely to have preexisting extramedullary hematopoietic paraspinal nodules and vascular disease than transfusion‐dependent patients, and preexisting increased risk for these complications must be kept in mind. Mitapivat is known to cause aromatase inhibition with an increase in some hormone levels, particularly testosterone, which must also be considered in specific situations. It may also result in an elevation of hepatic enzymes, which might necessitate close monitoring.^17^, ^18^

Thus, we are faced here with two agents that have unequivocal erythroid response, although at a slightly varying magnitude of effect, with one (mitapivat) clearly demonstrating benefit on functional outcomes. This does not fully dismiss luspatercept's effects on PRO as these seem to take a while to mature, especially in patients having erythroid response. Both agents would seem to be effective treatment options for anemia in adults with NTDT, with over half of the patients expected to achieve a response. However, additional data from real‐world evidence studies would be needed to better identify predictors of response that could help tailor management and choose one option over the other for the individual patient, based on their disease profile. Both agents seem to affect biomarkers of ineffective erythropoiesis (and hemolysis for mitapivat),^16^, ^17^, ^18^, ^20^ and whether these markers could help in patient selection for treatment merits evaluation. Anemia may only be a marker of the underlying ineffective erythropoiesis/hemolysis, and changes beyond those observed in hemoglobin level could help determine the extent of disease modification. This is important considering the mechanism of action of each agent. Luspatercept mainly affects SMAD2/3 signaling in the erythroid precursor maturation cascade and increases the output of red blood cells, and may have prosurvival effects on erythroid precursors. This has only been studied in β‐thalassemia, where ineffective erythropoiesis is more severe as compared to most forms of deletional hemoglobin H.^11^, ^12^, ^26^ Mitapivat has been shown to improve the efficiency of erythropoiesis, likely by allowing the developing precursor to better withstand oxidative stress from hemichrome formation, thus allowing it to mature rather than undergo apoptosis. It has also been shown to increase ATP production in the red cells produced, likely increasing their survival and reducing premature hemolysis.^9^, ^10^, ^15^ These mechanistic factors, patient genotype, and the degree of ineffective erythropoiesis and hemolysis may be important considerations in choosing a therapeutic option. Perhaps, in the future, combination trials may be undertaken to assess whether there may be any additive or synergistic effect of the two agents. Combinations with other drugs such as iron restriction agents have also shown beneficial effects for simultaneously improving both erythroid and iron parameters in preclinical models and warrant further investigation.^27^, ^28^

Additionally, the benefits of improving anemia extend beyond functional outcomes, and long‐term data are needed to assess the potential of reducing the incidence of disease‐related morbidity (including iron overload, assessed with liver iron concentration), especially if we are to advocate long‐term treatment.

The oral administration of mitapivat, compared to subcutaneous injections for luspatercept, which need to be given in a clinic, is a clear advantage especially for long‐term use and in NTDT patients who may not be frequent to treatment centers. Still, monitoring adherence to mitapivat in the real‐world setting may be needed as experience from oral iron chelation therapy in this patient population taught us that adherence could still be a challenge despite the convenience of administration. Of note, abrupt withdrawal has been associated with acute hemolysis in some patients in studies of mitapivat in pyruvate kinase deficiency, but no similar findings were reported in thalassemia.^29^

Luspatercept has also been evaluated and approved in patients with transfusion‐dependent thalassemia (BELIEVE trial) and myelodysplastic syndromes, and mitapivat has also been evaluated in a Phase 3 trial (ENERGIZE‐T trial) in patients with transfusion‐dependent thalassemia, while a similar pyruvate kinase activator, tebapivat, is being developed for myelodysplastic syndromes. Experience in indications other than NTDT can also help differentiate the roles of both drugs in targeting ineffective erythropoiesis. Lastly, the development of both agents in pediatric patients is eagerly awaited, since the detrimental impact of untreated anemia may have already manifested by the time patients move into adulthood. The capacity to prevent cumulative injury from the underlying disease will be an important differentiator of treatment value.

## AUTHOR CONTRIBUTIONS

All authors contributed to the manuscript drafting or critical review and final approval for submission.

## CONFLICT OF INTEREST STATEMENT

K.M.M. reports consultancy fees from Novartis, Bristol Myers Squibb (Celgene Corp), Agios Pharmaceuticals, CRISPR Therapeutics, Vifor Pharma, Novo Nordisk, and Pharmacosmos; research funding from Agios Pharmaceuticals and Pharmacosmos. S.S. reports consultancy fees from Agios Pharmaceuticals, Bristol Myers Squibb, and Novo Nordisk; being a member of a clinical trial steering committee for Vertex Pharmaceuticals; and research funding (for clinical trials) from Agios Pharmaceuticals, Bristol Myers Squibb, Novo Nordisk, and Regeneron. M.D.C. reports consultancy fees from Novartis, Bristol Myers Squibb (Celgene Corp), Vifor Pharma, and Vertex Pharmaceuticals; research funding from Novartis, Bristol Myers Squibb (Celgene Corp), La Jolla Pharmaceutical Company, Roche, Protagonist Therapeutics, and CRISPR Therapeutics. K.H.M.K. reports grants from Agios Pharmaceuticals and Pfizer; consulting fees from Agios Pharmaceuticals, Alexion Pharmaceuticals, Biossil, Bristol Myers Squibb, Forma, Novo Nordisk, Pfizer, and Vertex Therapeutics; honoraria from Agios Pharmaceuticals and Bristol Myers Squibb; and being on a data safety monitoring board/advisory board for Sangamo. A.K. reports receiving consulting fees/honoraria from Agios Pharmaceuticals, Bristol Myers Squibb (Celgene Corp), Chiesi Farmaceutici, CRISPR Therapeutics/Vertex, CSL Vifor, Novartis, and NovoNordisk; research support from Bristol Myers Squibb (Celgene Corp) and Novartis. Y.A. reports consultancy fees from Bristol Myers Squibb (Celgene Corp), CRISPR/Vertex and Silence Therapeutics; research funding from Agios, Bristol Myers Squibb (Celgene Corp), Cerus, Sobi Inc. and Novartis; and membership on an advisory committee for Cerus, Bristol Myers Squibb (Celgene Corp), Agios and Chiesi. V.V. reports grants from Agios Pharmaceuticals, Bristol Myers Squibb (Celgene Corp), DisperSol Technologies, IONIS Pharmaceuticals, Novartis, Pharmacosmos, The Government Pharmaceutical Organisation, and Vifor; consulting fees from Agios Pharmaceuticals, Bristol Myers Squibb (Celgene Corp), DisperSol Technologies, IONIS Pharmaceuticals, Novartis, Pharmacosmos, and Vifor. A.T.T. reports consultancy fees from Novo Nordisk, Bristol Myers Squibb (Celgene Corp), Agios Pharmaceuticals, Pharmacosmos, and Roche; research funding from Novo Nordisk, Bristol Myers Squibb (Celgene Corp), Agios Pharmaceuticals, Pharmacosmos, and Roche.

## FUNDING

No funding was received for this publication.

## Data Availability

Data sharing is not applicable to this article as no new data were created or analyzed in this review.
